# Disease burden and economic impact of diagnosed non-alcoholic steatohepatitis (NASH) in the United Kingdom (UK) in 2018

**DOI:** 10.1007/s10198-020-01256-y

**Published:** 2021-03-22

**Authors:** Alice Morgan, Sally L. Sansom, Emmanuel Tsochatzis, Philip N. Newsome, Stephen D. Ryder, Rachel Elliott, Lefteris Floros, Richard Hall, Victoria Higgins, George Stanley, Sandrine Cure, Sharad Vasudevan, Lynne Pezzullo

**Affiliations:** 1 Deloitte, Canberra, Australia; 2 Deloitte, Victoria, Australia; 3 UCL Institute for Liver and Digestive Health, Royal Free Hospital, London, UK; 4 National Institute for Health Research Biomedical Research Centre, University Hospitals Birmingham NHS Foundation Trust and the University of Birmingham, Birmingham, UK; 5 Centre for Liver and Gastrointestinal Research, Institute of Immunology and Immunotherpay, University of Birmingham, Birmingham, UK; 6 Liver Unit, University Hospitals Birmingham NHS Foundation Trust, Birmingham, UK; 7 National Institute for Health Research Nottingham Biomedical Research Centre at Nottingham University Hospitals NHS Trust and the University of Nottingham, Nottingham, UK; 8 University of Manchester, Manchester, UK; 9 PHMR Limited, London, UK; 10 Liver4Life, Bournemouth, United Kingdom; 11 Adelphi Real World, Cheshire, United Kingdom; 12 Intercept Pharmaceuticals, London, United Kingdom

**Keywords:** Cost-of-illness analysis, Non-alcoholic steatohepatitis (NASH), Burden of disease, Economic impact, Health care resource utilisation, I11, I12, I18, I39

## Abstract

**Background and aims:**

Non-alcoholic steatohepatitis (NASH) – a progressive subset of non-alcoholic fatty liver disease (NAFLD) – is a chronic liver disease that can progress to advanced fibrosis, cirrhosis, and end-stage liver disease (ESLD) if left untreated. Early-stage NASH is usually asymptomatic, meaning a large proportion of the prevalent population are undiagnosed. Receiving a NASH diagnosis increases the probability that a patient will receive interventions for the purpose of managing their condition. The purpose of this study was to estimate the disease burden and economic impact of diagnosed NASH in the United Kingdom (UK) adult population in 2018.

**Methods:**

The socioeconomic burden of diagnosed NASH from a societal perspective was estimated using cost-of-illness methodology applying a prevalence approach. This involved estimating the number of adults with diagnosed NASH in the UK in a base period (2018) and the economic and wellbeing costs attributable to diagnosed NASH in that period. The analysis was based on a targeted review of the scientific literature, existing databases and consultation with clinical experts, health economists and patient groups.

**Results:**

Of the prevalent NASH population in the UK in 2018, an estimated 79.8% were not diagnosed. In particular, of the prevalent population in disease stages F0 to F2, only 2.0% (F0), 2.0% (F1) and 16.5% (F2), respectively, were diagnosed. Total economic costs of diagnosed NASH in the UK ranged from £2.3 billion (lower prevalence scenario, base probability of diagnosis scenario) to £4.2 billion (higher prevalence scenario, base probability of diagnosis scenario). In 2018, people with NASH in the UK were estimated to experience 94,094 to 174,564 disability-adjusted life years (DALYs) overall. Total wellbeing costs associated with NASH in 2018 were estimated to range between £5.6 to £10.5 billion.

**Conclusion:**

The prevention and appropriate management of adult NASH patients could result in reduced economic costs and improvements in wellbeing.

**Supplementary Information:**

The online version contains supplementary material available at 10.1007/s10198-020-01256-y.

## Introduction

Non-alcoholic fatty liver disease (NAFLD) is a chronic liver disease characterised by excessive fat deposition in the liver that is not primarily attributable to consumption of alcohol and occurs in the absence of competing liver disease aetiologies such as chronic viral hepatitis [1, 2]. NAFLD is a major cause of chronic liver disease globally and it is emerging as the most common cause of abnormal serum aminotransferase levels as well as chronic liver disease [2, 3]. NAFLD is the hepatic manifestation of metabolic syndrome and is characterised by insulin resistance, Type 2 Diabetes Mellitus (T2DM), central (truncal) obesity, hyperlipidaemia and hypertension [4]. Non-alcoholic steatohepatitis (NASH) is a progressive subset of NAFLD. NASH is defined via liver biopsy as the presence of ≥ 5% hepatic steatosis and inflammation with hepatocyte injury (e.g., ballooning), with or without any fibrosis [5].

The number of NASH cases is projected to increase significantly in the near future, with estimates [6] suggesting a prevalence of NAFLD in a selection of European countries (France, Germany, Italy, Spain and the UK) of between 23.6% to 29.5% by 2030. Furthermore, the proportion of liver transplants performed in these same countries due to NASH has increased from an estimated 1.2% in 2002 to 8.4% in 2016 [7]. As such, NASH is on a trajectory to become the most common indication for liver transplantation in Europe – a trend which has similarly been observed in the United States (US) [5, 7].

Due to the practical and ethical difficulties involved in screening large samples of the population for NASH, limited evidence exists regarding the epidemiology and treatment patterns of NASH, and hence its associated costs over time. NASH has an economic cost through its effect on health, productivity, carers, efficiency of government administration and payments, and other economic costs. Furthermore, NASH has an adverse effect on wellbeing through a reduction in healthy life years lived. Therefore, a comprehensive examination of these economic and wellbeing costs is warranted.

The purpose of this study was to estimate the disease burden and economic impact of diagnosed NASH in the UK adult population in 2018. The development of such research may help to foster public health measures to tackle this important healthcare challenge. Increasing the proportion of NASH patients in the early stages of fibrosis (F0 to F2) with a diagnosis would provide opportunities for intervention to prevent progression to late stage disease.

## Methods

**Fig. 1:**
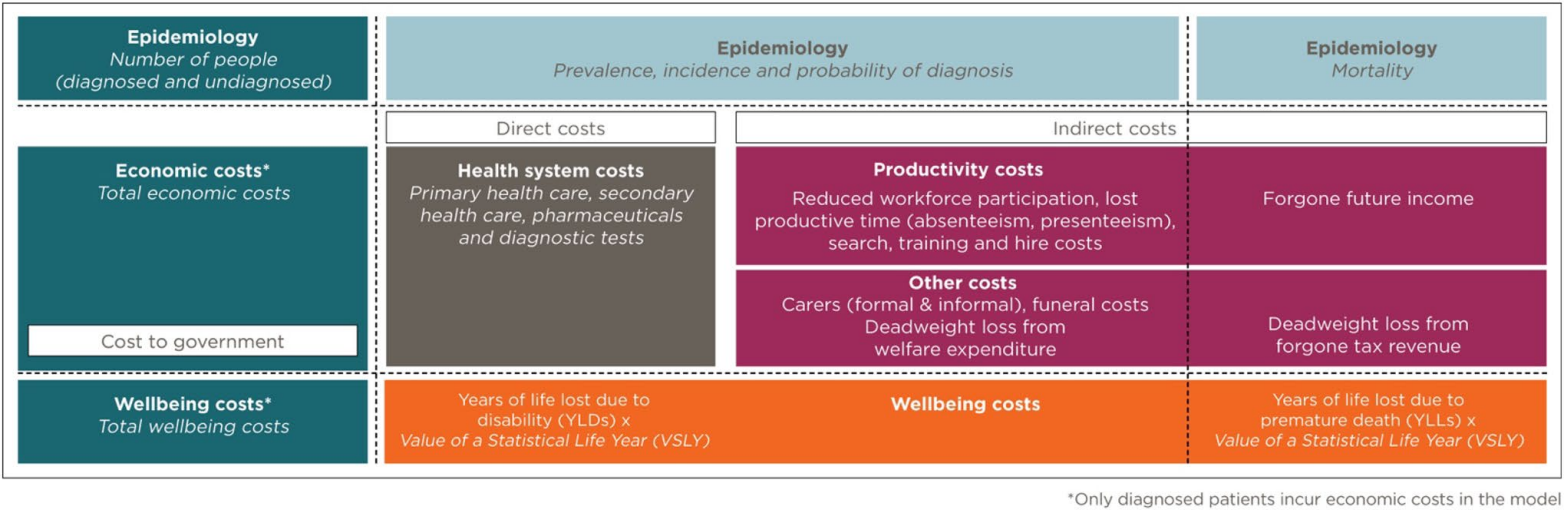
Model overview

The socioeconomic burden of diagnosed NASH in the UK was estimated from a societal perspective using cost-of-illness methodology applying a prevalence approach [8] via Excel (Fig. 1). The analysis was based on a targeted review of the scientific literature, existing databases and consultation with an expert panel comprising three clinical experts, two health economists and two patient group representatives. This involved estimating the number of people with diagnosed NASH in a base period (2018) and the economic and wellbeing costs attributable to this condition during the base year. The epidemiological inputs included in this estimation were the: population prevalence rate (lower and higher scenarios), prevalence distribution by age; sex; and disease stage, incidence, probability of diagnosis by disease stage (lower, base and higher scenarios), attributable liver transplants, and attributable mortality due to NASH. Probability of diagnosis was estimated due to the relationship between receiving a NASH diagnosis and the likelihood that a person would undergo management of their condition. As such, if a person is not diagnosed with NASH, it is assumed that their condition will not be managed [9]. Therefore, only patients diagnosed with NASH incur costs in the model. However, even though these patients are not monitored for their liver disease, this does not mean that these patients may not be managed or followed up by other healthcare specialists for their comorbidities. Despite this, costs attributable to comorbidities associated with NASH were excluded to facilitate estimating NASH-specific costs only.

Economic costs estimated include health system costs, productivity costs, formal and informal care costs, deadweight loss (DWL) from inefficiencies associated with forgone taxation revenue and transfer payments, and other economic costs such as funeral costs brought forward due to premature mortality. Economic costs were estimated via a combination of bottom-up and top-down approaches. Disease burden and wellbeing costs were estimated using World Health Organization (WHO) burden of disease methodology [10] and converted into pounds using an estimate of the value of a statistical life year (VSLY) [11].

The data available to estimate the socioeconomic burden of diagnosed NASH was limited and imperfect, requiring a considered methodological approach in the selection of inputs for the modelling. A targeted review of the scientific literature was conducted to obtain inputs for modelling (Supplementary Table 1.1), supplemented with information obtained from existing databases and through consultation with clinical experts, health economists, and patient groups. The review involved a targeted (non-systematic) search of the PubMed and Cochrane Library databases supplemented by hand searching. The review searched for publications released from 2011 onwards and in the English language. Relevant articles found at the title/abstract level, where the full text was not published in English were flagged. The selection of inputs for this study followed a structured, hierarchical approach considering three factors – quality, generalisability and internal consistency. NASH-specific UK studies were used, where available. Otherwise, each available input was considered in terms of its associated quality, generalisability and internal consistency trade-offs. The selection of a particular source; therefore, embodied a degree of judgement. Furthermore, where more than one methodology was available for estimating any of the inputs included in this analysis, consideration was given to its suitability to the overarching intent of this analysis and the available data in selecting the included methodology.

The inputs and methodologies selected were validated by the aforementioned expert panel via a series of consultations leveraging principles of expert elicitation [12, 13]. Inputs relating to the probability of diagnosis and health system utilisation of NASH patients underwent stepwise validation given the lack of available peer-reviewed literature and published data. The outcome of this process provided consensus regarding the clinical practice (diagnosis and management) of NASH patients in the UK in 2018. This process is further described in the ‘Diagnosis of NASH’ section.

### Epidemiology of NASH

NASH prevalence estimates (lower and higher scenarios) according to age, sex, and disease stage, and other epidemiological parameters including incidence, attributable liver transplants and attributable mortality were obtained from peer-reviewed literature and other published data (described below). Nine disease stages were included in the analysis – fibrosis stage zero (F0), fibrosis stage one (F1), fibrosis stage two (F2), fibrosis stage three (F3), fibrosis stage four compensated cirrhosis (F4 CC), decompensated cirrhosis (DCC), hepatocellular carcinoma (HCC), liver transplant, and death (including both liver- and cardiovascular-related death) [6].

An upper scenario estimate of the total prevalence rate of NASH in the adult population of the UK was derived from peer-reviewed literature [6]. The adult population prevalence of NASH in the UK in 2016 (4.1%) was assumed to be representative of the prevalence rate in 2018. Lower scenario sensitivity analysis was conducted by applying an alternate published modelled prevalence rate to the disease stage, age and gender distribution of NASH of 2.2% [2]. Age distribution was based on a US modelling study [14] for all disease stages, scaled to the overall prevalence. Sex distribution was based on the NAFLD population [15]. These prevalence rates were applied to population estimates by age and gender from the UK Office of National Statistics [16]. The incidence of NASH by disease stage was used to derive the newly diagnosed and monitored populations, assuming a proportionate diagnosis rate [17].

Liver transplants attributable to NASH were derived by applying the number of adult elective liver transplants in the UK due to the ‘metabolic’ indication, to the age and sex distribution of NASH prevalence [18, 19]. People aged 70 and over were assumed to be precluded from receiving a liver transplant [9]. This assumption was validated with local experts. Liver-related and excess cardiovascular disease (CVD) mortality was based on a previous study [6]. Excess CVD mortality was estimated to comprise 60% of total attributable mortality [6]. While the additional risk of CVD in NASH is contentious, it was included in this analysis following a review of the available evidence at the time of writing. The overall mortality due to NASH in the UK in 2016 was estimated to be 12,110 persons, or 0.023% of the adult population, which was assumed to be representative of the attributable mortality rate in 2018. This estimate was greater than the number of deaths attributable to NAFLD in 2016 to 2018 (732 deaths in total) reported by the UK Government; however, this source is limited in that it relies on accurate diagnostic coding [20].

### Diagnosis of NASH

The diagnosis rate of NASH by disease stage was determined based on consultation with clinical experts, highlighting that NASH diagnoses are primarily made incidentally during routine clinical investigations in the UK after patients are referred by their general practitioner (GP) to a liver specialist. It was suggested that a large proportion of the prevalent NASH population are undiagnosed prior to developing advanced fibrosis or cirrhosis. This is important as disease-specific intervention can only be offered after NASH diagnosis has been established. In the absence of a NASH diagnosis, no disease modifying interventions will be offered.

The probability of diagnosis at each disease stage was initially derived from a UK economic evaluation of an alternative diagnostic pathway, which reported the probability of detection from a UK prospective cross-sectional feasibility study [10–13]. In the UK economic evaluation cited, standard care is defined as referral to hospital for specialist investigation following an abnormal liver function test or other features, such as jaundice. The probability of detection via standard care was derived from a UK prospective cross-sectional feasibility study of the proposed alternative diagnostic pathway [13]. Individual patient data from the feasibility study were used by the aforementioned UK economic evaluation authors to generate input parameters related to target population characteristics and diagnostic effectiveness.

The estimates were presented and validated with a panel of clinical experts via a series of consultations leveraging principles of expert elicitation and amended to reflect local clinical practice. This involved presenting the panel with the estimated number of prevalent and diagnosed patients using the aforementioned sources. The panel drew from their knowledge of the number of diagnosed patients within their collective care and the number and size of specialist centres in the UK to provide feedback on the estimates. Estimates of the number of diagnosed patients with end stage liver disease (ESLD) were further validated against published estimates where available. Revised estimates were shared with the panel following their feedback for validation. This Delphi-type technique was repeated until consensus was achieved. These derived estimates are applied throughout the results section (base probability of diagnosis scenario). Sensitivity analysis on the probability of diagnosis was conducted via lower and higher scenarios. The results of the base scenario applied to the lower and higher prevalence scenarios are reported. The supplementary materials report the results of these lower and higher probability of diagnosis scenario applied to the lower and higher prevalence scenarios (Supplementary Tables 1.2–1.7).

### Health system costs of diagnosed NASH

Health system costs estimated include primary care, secondary care, diagnostic tests, pharmaceuticals and medical research. Health system utilisation data for patients diagnosed with NASH in disease stages F0 to HCC were extracted from two cost utility studies, containing information on NASH patient management and resource utilisation in the UK [9, 21]. These studies estimated health system utilisation using evidence from scientific literature and clinical practice guidelines from the National Institute for Health and Care Excellence (NICE), European Association for the Study of the Liver (EASL) and the American Association for the Study of Liver Disease (AASLD) [9, 21]. These sources were supplemented by the authors of the cited studies with expert opinion sought from a panel of regional UK liver specialists to accurately reflect how NASH patients were diagnosed and monitored in the UK [9]. Total health system costs in the initial and subsequent year following a liver transplant were extracted from studies of the costs of liver transplantation in the UK [22, 23].

Health system utilisation for patients in F0 to HCC was estimated for patients who were ‘newly diagnosed’ and ‘in monitoring’ in 2018. Newly diagnosed refers to patients who, in 2018, are in their first year of care following diagnosis. In monitoring are those patients who, in 2018, are in their second or subsequent years of care, following a previous diagnosis. Health system unit costs were sourced from the National Schedule of Reference Costs [24], National Tariff Payment System [25, 26], British National Formulary (BNF) [27] and Personal Social Services Research Unit (PSSRU) [28].

Total health system costs by disease stage were calculated by applying the average, per-person health system cost for that disease stage to the respective diagnosed population. The costs of treating comorbidities associated with NASH were not captured in this study, as the comorbidities are separate conditions. The distribution of costs across payers – such as individuals and families, government, and other parties (for example, private health insurers) – was estimated by applying proportions of healthcare expenditure proportions sourced from UK Health Accounts [29]. All costs derived from the literature were inflated to 2018 [28].

### Productivity costs of diagnosed NASH

Productivity costs were estimated via a human capital approach and include reduced workforce participation, lost productive time due to absenteeism and presenteeism, forgone income due to premature mortality, and search; hiring and training costs. This approach involves estimating the number of hours of productivity that are lost due to NASH. This estimate is then converted to a monetary value by multiplying the number of hours by average weekly earnings adjusted for age, gender, and general population employment rates [30]. The impact of premature death on workforce participation is captured by forgone future income.

Productivity costs incurred through reduced workforce participation were estimated by applying reduced workforce participation attributable to NASH to the UK general population employment rates and average weekly earnings by age and gender. In lieu of a UK-specific estimate, reduced workforce participation attributable to ESLD comprising DCC to liver transplant was obtained from a cross-sectional analysis of 230,406 adult US Medical Expenditure Panel Survey (MEPS) participants with chronic liver disease including NAFLD and NASH [31]. Similarly, costs incurred through absenteeism and/or presenteeism were estimated by multiplying the average number of weeks of productive time lost by average weekly earnings. Absenteeism and presenteeism estimates for disease stages F0 to liver transplant were informed by a retrospective analysis of the Adelphi NASH Disease Specific Programme™ (DSP), a large, multinational, point-in-time survey of physicians and their patients in a real-world clinical setting conducted from January through June 2018 in the five European countries. 296 physicians (139 hepatologists and 157 gastroenterologists) provided data for 2,060 NASH patients and the methodology has been described in detail and validated previously [32, 33]. Patients were eligible if they were over 18 years, had a physician-confirmed NASH diagnosis (via liver biopsy or non-invasive test approach) and were not participating in a clinical trial at the time of the survey. Physicians completed record forms for seven patients presenting for routine care capturing clinical details including tests conducted and associated values. Patients were also invited to complete a voluntary self-reported questionnaire including the Work Productivity and Activity Impairment (WPAI) validated measure [34]. Of the 2060 patients included in the NASH DSP, 724 patients qualified for analysis with physician-reported clinical test values for fibrotic assessment and a corresponding patient-reported questionnaire capturing WPAI responses. Retrospective WPAI analysis from the NASH DSP included the following: the data set included patients with disease stages F0 to F4 CC, hence estimates from disease stage F4 CC were extrapolated to include DCC, HCC and liver transplant disease stages; patients with type 2 diabetes were removed; patients were classified by F-stage severity (F0-F1, F2, F3, F4) via retrospective clinical assessment based on clinical test values (early fibrosis, indeterminate fibrosis, advanced fibrosis) to ensure correct severity classification. The NASH DSP obtained ethics approval from Freiburg Ethics Commission International (FEKI; Approval No. 017/1931) for five European countries [33]. All patients provided written informed consent for use of their aggregated data [33].

### Other economic costs of diagnosed NASH

Other economic costs of NASH include formal and informal care costs, DWL from inefficiencies associated with forgone taxation revenue and transfer payments, and other economic costs such as funeral costs brought forward due to premature mortality.

The average cost of formal care received by NASH patients with ESLD was based on a retrospective cost-of-illness study of patients with chronic liver disease conducted over 1 year in Italy, adjusted to 2018 UK pounds sterling [35]. The opportunity cost method was used to estimate the cost of informal care. This method measures the value of the alternative use of time spent caring, which is typically valued by productivity losses (or value of leisure time) associated with caring. It is based on the assumption that time spent providing informal care could be alternatively used within the paid workforce or in leisure activities. The proportion of NASH patients with ESLD who received informal care was obtained from peer-reviewed literature [35]. The average time spent on informal care in the UK was calculated using the Family Resources Survey (FRS) [36]. Informal care requirements were assumed to apply evenly across age and gender, varying only by disease stage. The age and gender adjusted average weekly earnings of primary carers in the UK was obtained from published data [36].

DWL was estimated from inefficiencies associated with forgone taxation revenue and transfer payments [37]. Transfer payments estimated include government expenditure on healthcare and welfare. The following welfare payments were estimated for NASH patients with ESLD: Personal Independence Payment (PIP), Employment and Support Allowance (ESA) and Carer’s Allowance (CA). The number and value of PIP claims each year attributable to NASH was calculated using administrative data from the Department of Work and Pensions on the number of PIP claimants with liver disease, adjusted for the proportion of total liver disease in the UK which is due to NASH [38, 39]. The number and value of ESA claims attributable to NASH each year was calculated using administrative data from published data [40] on the number of ESA claimants, adjusted for the percentage of the UK population with NASH. To estimate the number and value of CA claims due to NASH, the proportion of people who require care and have a carer who receives CA (for all conditions) was applied to the eligible population and rate of payment [41]. Funeral costs brought forward due to premature mortality were sourced from publicly available information adjusted to 2018 [42].

### Disease burden and wellbeing costs of diagnosed NASH

Disease burden and wellbeing costs were estimated using the WHO burden of disease methodology via Excel. This is a non-financial approach, where pain, suffering and premature mortality are measured in terms of Disability-Adjusted Life Years (DALYs).

DALYs are composed of premature mortality (years of life lost due to premature death – YLL) and morbidity (years of healthy life lost due to disability – YLD) components. DALYs are calculated by assigning disability weights to various health states, where zero represents a year of perfect health and one represents death. Disability weights for DCC and HCC were sourced from the Institute for Health Metrics and Evaluation (IHME) Global Burden of Disease Study [43]. A disability weight of 0.178 was applied to DCC, and a disability weight of 0 was applied to F4 CC. Several disability weights are available for HCC depending on the state of the disease (diagnosis, 0.29 to terminal, 0.54). Disability weights for HCC were weighted according to the proportion of time spent in each state, for an overall disability weight of 0.296 [43]. DALYs are discounted at a rate of 3% consistent with WHO methodology [44]. The authors of this cost-of-illness study also note the disability weight for DCC (mean: 0.163, 95% CI 0.136–0.194) estimated via web-based sample surveys in Hungary, Italy, the Netherlands and Sweden [45]. The DCC disability weight used in this cost-of-illness study falls within the range reported [45].

The burden of disease as measured in DALYs was converted into pounds using an estimate of the VSLY, an estimate of the value society places on an anonymous life. A VSLY of £60,000 per person in the UK was sourced from ‘The Green Book’ published by HM Treasury and inflated using the UK Health Consumer Price Index (CPI) [11].

### Sensitivity analysis

Sensitivity analysis was conducted in Excel on the population prevalence rate and probabilities of diagnosis (described above) and are reported in the supplementary materials. In addition, aggregate economic cost variables which were most likely to influence the results were adjusted from their base value to a high or low case (default ± 10%) at each disease stage to determine the magnitude of their influence on the results estimated.

## Results

### Diagnosed NASH population

**Fig. 2:**
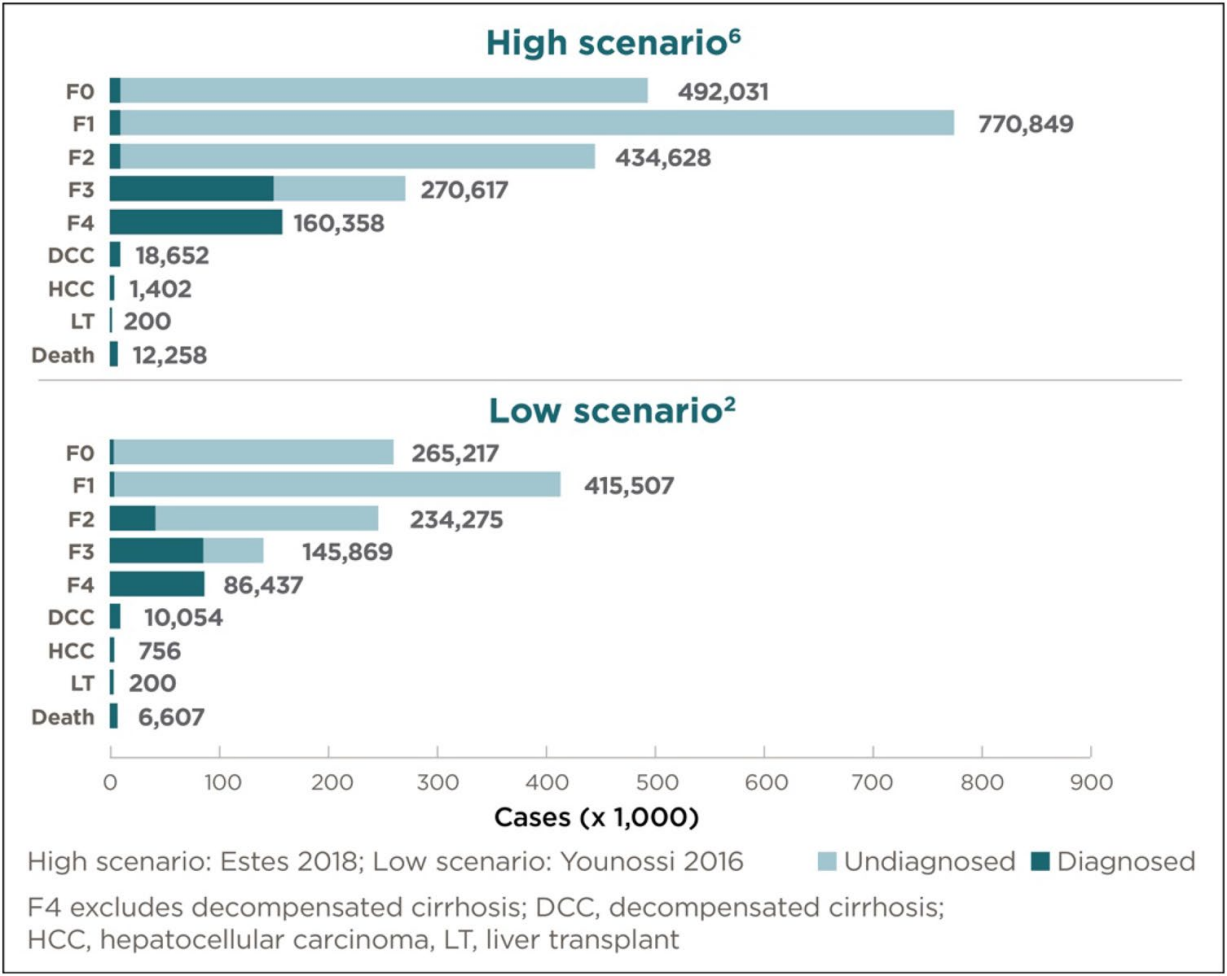
Estimated prevalent cases of NASH in the UK (2018) by probability of diagnosis and disease stage (base probability of diagnosis scenario)

Of the prevalent adult NASH population in the UK in 2018, those patients with a diagnosis ranged between 0.23 million persons (lower prevalence scenario, base probability of diagnosis scenario) to 0.44 million persons (higher prevalence scenario, base probability of diagnosis scenario) (Fig. 2; Tables 1 and 2). Of the prevalent proportion with advanced fibrosis and cirrhosis, 73.8% were diagnosed. By comparison, of the prevalent population in disease stages F0 to F2, only 2.0% (F0), 2.0% (F1) and 16.5% (F2), respectively, were diagnosed.

**Table 1:** Probability of diagnosis by disease stage (percentage, %) (base probability of diagnosis scenario)

Disease stage	F0	F1	F2	F3	F4 CC	DCC	HCC	LT
Probability of diagnosis (%) (base scenario)	2.0%	2.0%	16.5%	58.3%	100.0%	100.0%	100.0%	100.0%

*F0* Fibrosis stage zero, *F1* Fibrosis stage one, *F2* Fibrosis stage two, *F3* Fibrosis stage three, *F4 CC* Fibrosis stage four compensated cirrhosis, *DCC* Decompensated cirrhosis, *HCC* hepatocellular carcinoma, *LT* liver transplant

**Table 2:** Diagnosed population results* (base probability of diagnosis scenario)

	F0	F1	F2	F3	F4 CC	DCC	HCC	LT	Death	Total
*Higher prevalence scenario*										
Diagnosed (% of prevalence)	2.00	2.00	16.50	58.25	100.00	100.00	100.00	100.00	100.00	20.25
NASH diagnosed (millions)	0.01	0.02	0.07	0.16	0.16	0.02	–	–	0.01	0.44
*Lower prevalence scenario*										
Diagnosed (% of prevalence)	2.00	2.00	16.50	58.25	100.00	100.00	100.00	100.00	100.00	20.26
NASH diagnosed (millions)	0.01	0.01	0.04	0.08	0.09	0.01	–	–	0.01	0.23

*Some results are zero (indicated as -) due to rounding

*F0* Fibrosis stage zero, *F1* Fibrosis stage one, *F2* Fibrosis stage two, *F3* Fibrosis stage three, *F4 CC* Fibrosis stage four compensated cirrhosis, *DCC* Decompensated cirrhosis, *HCC* hepatocellular carcinoma, *LT* liver transplant

### Economic costs of diagnosed NASH

Total economic costs of diagnosed NASH in the UK were estimated to range between £2.3 to £4.2 billion in 2018.: £479 to £889 million (21%) of these costs were attributable to people with NASH in F3, while £553 to £1,026 million (24%) and £164 to £303 million (7%) were attributed to people with NASH F4 CC to DCC (Table 3). The economic cost of NASH was estimated to range between £9,420 to £9,450 per person with a diagnosis in 2018 (per person costs are slightly higher in the lower scenario as medical research costs are distributed over a smaller population) (Table 4).

**Table 3:** Economic costs results (total, £ million) (base probability of diagnosis scenario)

	F0	F1	F2	F3	F4 CC	DCC	HCC	LT	Death	Total
*Higher prevalence scenario*										
Health system costs	2	3	27	75	127	105	10	11	–	359
Productivity costs	47	74	342	776	860	171	13	2	1421	3707
Carer costs	–	–	–	–	–	23	2	–	–	25
Other economic costs	–	–	–	–	–	–	–	–	18	18
Deadweight loss	2	4	17	38	39	4	-	-	2	107
Total economic costs	51	80	387	889	1026	303	25	14	1441	4215
*Lower prevalence scenario*										
Health system costs	1	2	15	40	69	57	5	11	–	200
Productivity costs	25	40	185	418	464	92	7	2	766	1999
Carer costs	–	–	–	–	–	12	1	–	–	14
Other economic costs	–	–	–	–	–	–	–	–	10	10
Deadweight loss	1	2	9	21	21	2	–	–	1	58
Total economic costs	28	44	209	479	553	164	13	14	777	2280

*Some results are zero (indicated as -) due to rounding

*F0* Fibrosis stage zero, *F1* Fibrosis stage one, *F2* Fibrosis stage two, *F3* Fibrosis stage three, *F4 CC* Fibrosis stage four compensated cirrhosis, *DCC* Decompensated cirrhosis, *HCC* hepatocellular carcinoma, *LT* liver transplant

**Table 4:** Economic costs results (per person, £) (base probability of diagnosis scenario)

	F0	F1	F2	F3	F4 CC	DCC	HCC	LT	Death	Total
*Higher prevalence scenario*										
Health system costs	181	181	381	473	793	5623	6861	55,341	-	803
Productivity costs	4,775	4775	4775	4923	5363	9189	9189	11,943	115,961	8285
Carer costs	–	–	–	–	–	1212	1224	2293	–	55
Other economic costs	–	–	–	–	–	–	–	–	1449	40
Deadweight loss	241	241	241	241	241	241	241	309	145	238
Total economic costs	5,197	5197	5398	5637	6397	16,264	17,514	69,885	117,555	9420
*Lower prevalence scenario*										
Health system costs	223	223	386	474	794	5624	6862	55,341	–	828
Productivity costs	4775	4775	4775	4923	5363	9189	9189	11,943	115,961	8286
Carer costs	–	–	–	–	–	1212	1224	2293	–	56
Other economic costs	–	–	–	–	–	–	–	–	1449	40
Deadweight loss	243	243	243	243	243	243	243	312	147	240
Total economic costs	5242	5242	5405	5640	6000	16,267	17,517	69,889	117,557	9450

*Some results are zero (indicated as -) due to rounding

*F0* Fibrosis stage zero, *F1* Fibrosis stage one, *F2* Fibrosis stage two, *F3* Fibrosis stage three, *F4 CC* Fibrosis stage four compensated cirrhosis, *DCC* Decompensated cirrhosis, *HCC* hepatocellular carcinoma, *LT* liver transplant

If 100% of the prevalent NASH population was diagnosed, this cost-of-illness study estimated that total economic costs would have totalled £7.0 to £13.0 billion in 2018.

### Health system costs of diagnosed NASH

**Fig. 3:**
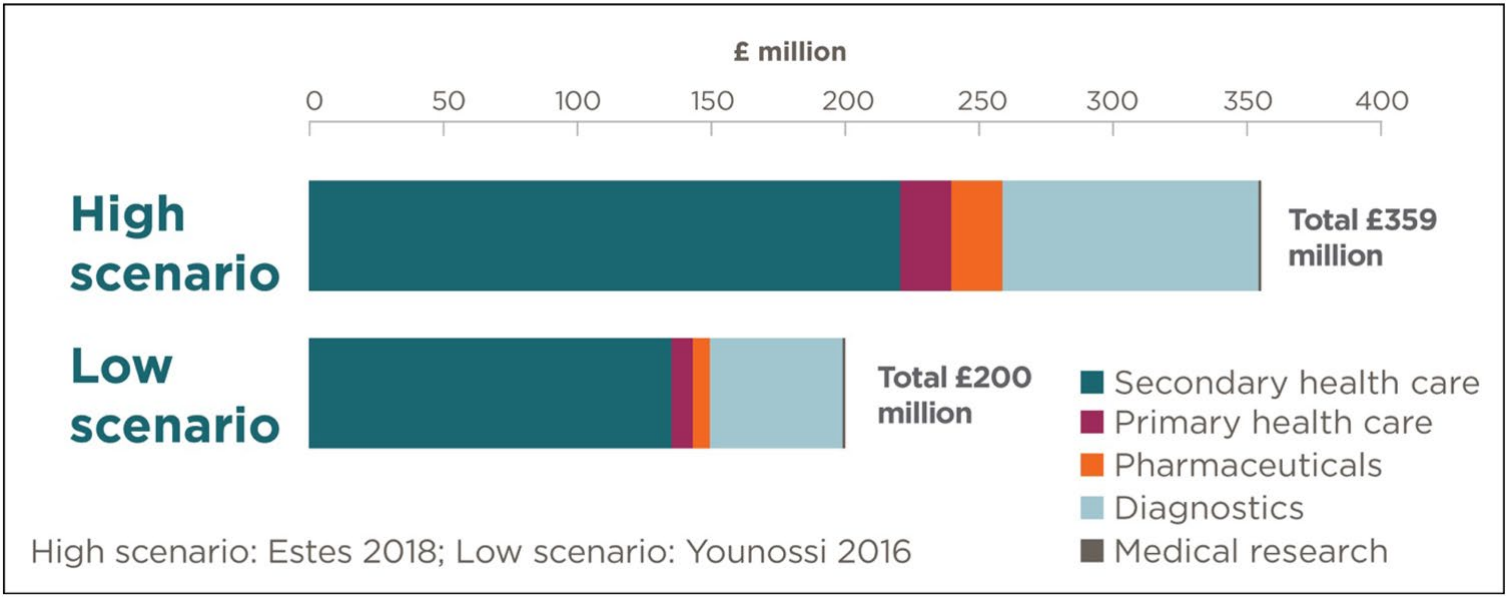
Total health system costs of all stage NASH by type (£ million) (base probability of diagnosis scenario)

Total health system costs due to diagnosed NASH were estimated to range between £200 to £359 million in 2018, with F4 CC incurring the greatest share of total health system costs at £69 to £127 million (35%) (Fig. 3). The average per patient cost was estimated to range between £803 to £828 (per person costs are slightly higher in the lower scenario as medical research costs are distributed over a smaller population). The majority of health system costs were incurred in secondary care (£126 to £224 million, 62%) or were attributed to diagnostic testing (£48 to £90 million, 25%). The UK government bore approximately 79% of health system costs, while individuals and families bore 15%, and other parties (such as private health insurers) bore 6% of health system costs.

### Productivity costs of diagnosed NASH

In 2018, the productivity cost of diagnosed NASH in the UK was estimated to range between £2.0 to £3.7 billion. People in F3 and F4 CC disease stages incurred the greatest productivity costs at 21% and 23% of total productivity costs, respectively. Per person, the average productivity cost across all disease stages of NASH was £8285 (both scenarios). The costs of forgone income made up the largest share of productivity costs at 58%. Reduced workforce participation, absenteeism and presenteeism made up 3%, 13% and 46% of total productivity costs, respectively. The government incurred 22% of total costs through lost taxes, and employers and individuals incurred 46% and 32% of costs, respectively.

### Other economic costs of diagnosed NASH

Overall, other economic costs attributable to diagnosed NASH were estimated to range between £81 to £149 million in 2018. This consists of carer costs of £14 to £25 million, DWL of £58 to £107 million and other economic costs (funeral costs) of £10 to £18 million. The total annual cost of formal and informal care for NASH patients was estimated to range between £13 to £24 million and £1 (both scenarios) million in 2018, respectively. Excluding LT, people with NASH in disease stage HCC incurred the greatest per person carer costs, estimated at £1224 (both scenarios) in 2018. DWL made up the majority of the other economic costs category ranging from £58 to £107 million. Since DWL is borne by the whole of society, the majority of ‘other costs’ were consequently also borne by society. The discounted value of funeral costs associated with premature deaths due to NASH was estimated to range between £10 to £18 million.

### Disease burden and wellbeing costs of diagnosed NASH

In 2018, people diagnosed with NASH in the UK were estimated to experience 94,094 to 174,564 DALYs overall. Total wellbeing costs associated with NASH in 2018 were estimated to range between £5.6 to £10.5 billion (Table 5). Per person, the wellbeing cost of NASH was approximately £23,400 in both scenarios (Table 6).

**Table 5:** Wellbeing costs results (total, £ million) (base probability of diagnosis scenario)

	F0	F1	F2	F3	F4 CC	DCC	HCC	LT	Death	Total
*Higher prevalence scenario*										
Total wellbeing costs	–	–	–	–	–	199	25	–	10,250	10,474
*Lower prevalence scenario*										
Total wellbeing costs	–	–	–	–	–	107	13	–	5525	5646

*F0* Fibrosis stage zero, *F1* Fibrosis stage one, *F2* Fibrosis stage two, *F3* Fibrosis stage three, *F4 CC* Fibrosis stage four compensated cirrhosis, *DCC* Decompensated cirrhosis, *HCC* hepatocellular carcinoma, *LT* liver transplant

**Table 6:** Wellbeing costs results (per person, £) (base probability of diagnosis scenario)

	F0	F1	F2	F3	F4 CC	DCC	HCC	LT	Death	Total
*Higher prevalence scenario*										
Total wellbeing costs	–	–	–	–	–	10,680	17,760	–	836,184	23,407
*Lower prevalence scenario*										
Total wellbeing costs	–	–	–	–	–	10,680	17,760	–	836,184	23,398

*F0* Fibrosis stage zero, *F1* Fibrosis stage one, *F2* Fibrosis stage two, *F3* Fibrosis stage three, *F4 CC* Fibrosis stage four compensated cirrhosis, *DCC* Decompensated cirrhosis, *HCC* hepatocellular carcinoma, *LT* liver transplant

## Discussion

Early-stage NASH is usually characterised by minimal symptomatology, resulting in a large proportion of the prevalent population thought to be undiagnosed in 2018. The severity of symptoms, and risk of liver-related illness and death increases with the severity of the disease stage. The rate of increase is most significant from disease stages F3 and F4 CC, onwards [46]. This substantiates the finding from this study that, of the prevalent population in disease stages F0 to F2, only 2.0% (F0), 2.0% (F1) and 16.5% (F2), respectively, were diagnosed. One of the reasons identified for this through discussion with local clinical experts is that general practitioners in the UK act as ‘gate-keepers’ to specialist medical practitioners such as hepatologists. This means patients are commonly referred to a hepatologist only once a NASH diagnosis, or liver-related concern more generally, is suspected. Once diagnosed, patients are more likely to receive interventions for the purpose of managing this condition. While early-stage NASH patients are commonly referred back to their general practitioner for monitoring and are recommended lifestyle modifications, patients with advanced fibrosis, cirrhosis or ESLD due to NASH are more likely to be referred, diagnosed and actively monitored by liver specialists. This is reflected in the proportion of diagnosed patients within each disease stage, in addition to the health system services and pharmaceuticals utilised within each stage. As such, this study found that the proportion of diagnosed patients and health system costs were greater in patients with ESLD than in earlier disease stages.

Of the prevalent NASH population in the UK in 2018, an estimated 79.8% were not diagnosed. The majority of this undiagnosed population had early-stage NASH which, if left untreated, may progress to more expensive ESLD in the coming years. As such, patients with early-stage NASH can be thought of as dormant cases, meaning they incur low costs in the present while having the potential to incur significant economic and wellbeing costs in the future. This is important, because the prevalence of NASH is forecast to increase significantly over time [6]. Effective management of early-stage NASH patients requires that they first be diagnosed. Increasing the proportion of early-stage NASH patients with a diagnosis will increase the health system services and pharmaceuticals accessed by those patients for the purpose of managing their condition. This would increase costs incurred by early-stage NASH patients in the present, with the intention of reducing costs incurred by these patients in the future.

In deciding how to proceed, the National Health System (NHS) first needs to understand whether improved diagnosis and management of early-stage NASH is a cost-effective approach to reducing the rate at which these patients progress to more expensive ESLD. It is not practical or ethical to screen an entire population for NASH; however, it is worth exploring whether screening patients at increased risk of progressing to ESLD (i.e., patients with metabolic syndrome) is cost-effective. This question was explored in a modelled cohort study of 1,000 NAFLD patients in the UK over 1 year from a healthcare payer perspective. This study found that, compared with standard care, non-invasive liver testing in primary care was cost-effective in supporting early detection of patients at risk of progressing to ESLD while reducing unnecessary patient referrals with early stage liver disease [47]. Furthermore, a UK cost-utility analysis of a community-based stratification pathway of patients at risk of NAFLD found this pathway demonstrated an 85% probability of cost-effectiveness compared with standard care at the UK willingness-to-pay threshold of £20,000 per QALY [9]. These approaches are supported by the NICE clinical guidelines which recommend testing people with NAFLD suspected of having advanced fibrosis [48]. Other strategies could include public health interventions, such as education and lifestyle modifications, targeting populations with metabolic syndrome whom are at increased risk of developing NASH [4]. As noted in previous studies [21], limitations in the available data prevent robust analysis of long-term care pathways for NAFLD and NASH patients [21, 49].

In light of global concern regarding NASH in the context of a worldwide epidemic of T2DM and obesity, it is important that diagnostic and management pathways for NASH be improved [2, 4, 6]. A particular topic of focus is identifying a suitable screening pathway for ensuring the most appropriate mix of patients are referred to specialists. The UK is leading the way in this regard, where studies of novel screening methods have been shown to be cost-effective [50, 51]. These studies involved screening patients via serum-based markers and determining their NASH diagnosis status via transient elastography in primary care, followed by referral of diagnosed patients only to hepatologists for treatment. These studies found that the novel screening pathway resulted in the identification of a significant number of new cirrhosis cases [50, 51]. This pathway is distinct from other pathways in that it involves non-invasive screening and diagnosis of patients in primary care, followed by referral to hepatologists for triage and treatment, as opposed to relying on serum-based screening in primary care followed by referral to hepatologists for diagnosis and treatment. Canada is another such country, where a team of hepatologists in Calgary, Alberta are studying a similar diagnostic/primary care pathway for NAFLD patients [52]. A study of this pathway is underway and is expected to be published in early 2020. Other efforts to improve the management of NASH patients include a US cost-utility analysis and deterministic Markov model comparing the costs and health benefits of lifestyle modification alone or with pioglitazone or vitamin E in a cohort of patients aged 50 years with biopsy proven F3 NASH or greater [53]. This cited study found that pharmacological treatment, including pioglitazone, combined with lifestyle modification is likely to be cost-effective [53].

Cost-of-illness studies are conducted with the intent of describing the economic burden imposed by a disease, on a specific population [8]. The results from cost-of-illness studies can be used to justify investment in preventive or treatment interventions, inform funding allocation and prioritisation, provide a basis for policy and planning, and provide inputs for economic analyses. This could include: cost benefit analyses (CBA), cost effectiveness analyses (CEA), cost utility analyses (CUA), and cost minimisation analyses (CMA) [54, 55]. This study fills an important gap in the literature by bringing together the best available evidence to provide an estimate of the economic and wellbeing cost of NASH in the diagnosed adult population in the UK in 2018, by disease stage [56–61]. The results from this cost-of-illness study could be used to inform the targeting of educational programs for the purpose of increasing the awareness of NASH and its associated risks. The results from this analysis could also provide inputs for screening and treatment (including preventive treatment) reimbursement decisions.

The targeted review of the scientific literature undertaken found one previously published estimate of the economic burden of NAFLD or NASH in the UK in 2016 [2]. This cited study estimated the cost of NAFLD and NASH in France, Germany, Italy, the UK and the US. The overall costs of NAFLD and NASH in the UK estimated [2] was £5.24 billion of economic (‘direct’) costs and £26.03 of billion wellbeing (‘societal’) costs (2016 lb sterling). In comparison to the current study, the cited study [2] estimated the economic burden of NAFLD and NASH using a Markov model of these populations in the UK. Health system costs were estimated for NAFLD patients. Productivity costs and other economic costs were not estimated. Wellbeing costs were estimated by applying the differential in the utility score associated with each disease state and to the UK willingness-to-pay threshold. The authors of the current study also note the work underway to publish the results from the Global Assessment of the Impact of NASH (GAIN) study estimating the socio-economic burden of NASH in Europe and the US. The authors were limited in their ability to compare their approach and results to those of the GAIN study, because the latter were not published at the time this manuscript was prepared.

Many of the epidemiological, economic and wellbeing parameters required to model the socioeconomic burden of NASH in the adult population in the UK in 2018 were supported by limited available evidence, providing estimates of these parameters for NASH in the UK, specifically. Due to these limitations, evidence was sourced from studies investigating conditions with comparable aetiology and countries with comparable demographics and validated via a series of consultations leveraging principles of expert elicitation [12, 13]. This means that in many cases the inputs underlying this study are uncertain, and changes in these inputs and parameters may have a significant impact upon the total estimate of the costs of NASH in the adult population in the UK in 2018. To assist in quantifying the impact of this uncertainty, a scenario analysis approach was used, whereby uncertain parameters were adjusted from their base value to a high or low case at each disease stage. As expected, the prevalence rate and probability of diagnosis were sensitive parameters. Specifically, NASH can only be officially diagnosed via liver biopsy. Given it is not feasible to conduct liver biopsies in studies of the general population (due to practical and ethical considerations), there is no direct assessment of the prevalence or incidence of NASH. Alternative measures such as advanced fibrosis may be a surrogate measure during the diagnostic investigation. Furthermore, there are limitations in applying US productivity data to the UK given the different factors driving employee behaviours. As a result, the productivity inputs used in this study are an estimation of the productivity effects attributable to NASH in the UK, noting the complexities in assigning attribution to NASH directly while excluding the effects of reduced productivity due to comorbidities such as obesity and diabetes. These represent areas for future research.

## Conclusion

This study found NASH imposed significant economic and wellbeing costs on the UK population in 2018. As it currently stands, people with NASH incur significant economic costs, in addition to severe reductions in their quality of life. The greatest economic burden is estimated to be borne by individuals diagnosed with NASH; however, significant economic costs are also incurred by families, friends, the government, employers and society/others.

These results demonstrate the need to develop effective non-interventional diagnoses and treatments for NASH in addition to preventative measures. Educational programs aimed at increasing awareness of NASH, and associated risks, among general practitioners and the general population will be important for reducing the rate of progression from early to late stage liver disease. This is important, because the late, fibrotic stages of NASH are estimated to incur the greatest economic and wellbeing costs to individuals and others.

## Supplementary Information

Below is the link to the electronic supplementary material.

Supplementary file1 (DOCX 201 KB)

## Data Availability

The data sets supporting the results of this article are available to the public from various government sources. All data sets relied upon have been cited, where appropriate, in the manuscript and included in the reference list. A full report which outlines all methods and data sources, along with detailed results, is available on request.

## Abbreviations

CA: Carer’s allowance
CC: Compensated cirrhosis
CBA: Cost benefit analyses
CEA: Cost effectiveness analyses
CMA: Cost minimisation analyses
CUA: Cost utility analyses
CVD: Cardiovascular disease
DALY: Disability-adjusted life year
DCC: Decompensated cirrhosis
DSP: Disease Specific Programme™
DWL: Deadweight loss
ESA: Employment and support allowance
F0-DCC: Fibrosis stages which include measurement of the amount of liver fibrosis:
F0: Fibrosis stage zero
F1: Fibrosis stage one
F2: Fibrosis stage two
F3: Fibrosis stage three
F4 CC: Fibrosis stage four compensated cirrhosis
DCC: Decompensated cirrhosis
FRS: Family Resources Survey
GAIN: Global Assessment of the Impact of NASH
HCC: Hepatocellular carcinoma
HCV: Hepatitis C virus
HM Treasury: Her Majesty’s Treasury
ICD: International Classification of Diseases
IHME: Institute for Health Metrics and Evaluation
LT: Liver transplant
MEPS: Medical Expenditure Panel Survey
NAFLD: Non-alcoholic fatty liver disease
NASH: Non-alcoholic steatohepatitis
NHS: National Health System
NICE: National Institute for Health and Care Excellence
PIP: Personal independence payment
PRO: Patient reported outcome
PSSRU: Personal Social Services Research Unit
T2DM: Type 2 diabetes mellitus
UK: United Kingdom
US: United States
VSLY: Value of a statistical life year
WHO: World Health Organization
WPAI: Work productivity activity index
YLD: Years of healthy life lost due to disability
YLL: Years of life lost due to premature death
