# Supplementary material for: Disease burden and economic impact of diagnosed non-alcoholic steatohepatitis (NASH) in the United Kingdom (UK) in 2018
Source: Eur J Health Econ. 2021 Mar 22;22(4):505–18. doi: 10.1007/s10198-020-01256-y (PMC8166804; doi:10.1007/s10198-020-01256-y)
Supplement: Supplementary file 1 — Supplementary file1 (DOCX 201 KB) [file 10198_2020_1256_MOESM1_ESM.docx]

Disease burden and economic impact of diagnosed non-alcoholic steatohepatitis (NASH) in the United Kingdom (UK) in 2018

# Authors

Alice Morgan^1^, Sally Hartmanis^2^, Emmanuel Tsochatzis^3^, Philip N. Newsome^4–6^, Stephen D. Ryder^7^, Rachel Elliott^8^, Lefteris Floros^9^, Richard Hall^10^, Victoria Higgins^11^, George Stanley^12^, Sandrine Cure^12^, Sharad Vasudevan^2^, Lynne Pezzullo^1^

**Affiliations and addresses**

1. Deloitte, Canberra, Australia; 2. Deloitte, Victoria, Australia; 3. UCL Institute for Liver and Digestive Health, Royal Free Hospital, London, United Kingdom; 4. National Institute for Health Research Biomedical Research Centre, University Hospitals Birmingham NHS Foundation Trust and the University of Birmingham, Birmingham, United Kingdom; 5. Centre for Liver and Gastrointestinal Research, Institute of Immunology and Immunotherpay, University of Birmingham, Birmingham, United Kingdom; 6. Liver Unit, University Hospitals Birmingham NHS Foundation Trust, Birmingham, United Kingdom; 7. National Institute for Health Research Nottingham Biomedical Research Centre at Nottingham University Hospitals NHS Trust and the University of Nottingham, Nottingham, United Kingdom; 8. University of Manchester, Manchester, United Kingdom; 9. PHMR Limited, London, United Kingdom; 10. Liver4Life, Bournemouth, United Kingdom; 11. Adelphi Real World, Cheshire, United Kingdom; 12. Intercept Pharmaceuticals, London, United Kingdom

# Contact details

Alice Morgan

[alicemorgan@deloitte.com.au](mailto:alicemorgan@deloitte.com.au)

# Supplementary materials

## Cost-of-illness inputs

Cost-of-illness inputs

| **Parameter** | **Unit** | **General** | **F0** | **F1** | **F2** | **F3** | **F4 CC** | **DCC** | **HCC** | **LT** | **Source** |
| --- | --- | --- | --- | --- | --- | --- | --- | --- | --- | --- | --- |
| **Epidemiology** |  |  |  |  |  |  |  |  |  |  |  |
| Prevalence (higher scenario) | % | 4.1% |  |  |  |  |  |  |  |  | [1] |
| Prevalence (lower scenario) | % | 2.2% |  |  |  |  |  |  |  |  | [2] |
| Mortality (liver-related) | % | 0.009% |  |  |  |  |  |  |  |  | [1] |
| Mortality (CVD) | % | 0.014% |  |  |  |  |  |  |  |  | [1] |
| Distribution of prevalent population by disease stage | % |  | 22.9% | 35.9% | 20.2% | 12.6% | 7.5% | 0.9% | 0.1% |  | [3] |
| New cases as a proportion of total cases | % |  | 4% | 4% | 4% | 4% | 4% | 29% | 51% | 50% | [3] |
| Sex (percentage of cases that are male) | % | 56.1% |  |  |  |  |  |  |  |  | [4] |
| Number of liver transplants (2018) | number | 100 |  |  |  |  |  |  |  |  | [5, 6] |
| Number of liver transplants (2017) | number | 100 |  |  |  |  |  |  |  |  | [5, 6] |
| Percentage of cases diagnosed (base case) | % |  | 2% | 2% | 17% | 58% | 100% | 100% | 100% | 100% | Consultation, [7, 8] |
| Percentage of cases diagnosed (lower scenario) | % |  | 1% | 1% | 1% | 1% | 2% | 95% | 100% | 100% | Consultation, [7, 8] |
| Percentage of cases diagnosed (higher scenario) | % |  | 10% | 15% | 17% | 58% | 80% | 100% | 90% | 100% | Consultation, [7, 8] |
| Percentage of cases diagnosed (complete scenario) | % |  | 100% | 100% | 100% | 100% | 100% | 100% | 100% | 100% | Consultation, [7, 8] |
| **Health system costs** |  |  |  |  |  |  |  |  |  |  |  |
| Primary care (newly diagnosed) | £/year |  | 37 | 37 | 37 | 37 | 37 | 149 | 0 | 0 | Consultation, [7, 8, 9, 10, 11, 12, 13, 14] |
| Primary care (in monitoring) | £/year |  | 75 | 75 | 75 | 37 | 37 | 149 | 0 | 0 | Consultation, [7, 8, 9, 10, 11, 12, 13, 14] |
| Secondary care (newly diagnosed) | £/year |  | 461 | 461 | 461 | 595 | 708 | 4,440 | 5,743 | 92,909 | Consultation, [7, 8, 9, 10, 11, 12, 13, 14] |
| Secondary care (in monitoring | £/year |  | 0 | 0 | 188 | 305 | 351 | 4,456 | 3,970 | 17,771 | Consultation, [7, 8, 9, 10, 11, 12, 13, 14] |
| Pharmaceuticals (newly diagnosed) | £/year |  | 23 | 23 | 23 | 23 | 41 | 344 | 1,985 | 0 | Consultation, [7, 8, 9, 10, 11, 12, 13, 14] |
| Pharmaceuticals (in monitoring | £/year |  | 23 | 23 | 23 | 23 | 36 | 369 | 1,985 | 0 | Consultation, [7, 8, 9, 10, 11, 12, 13, 14] |
| Diagnostic tests (newly diagnosed) | £/year |  | 233 | 233 | 564 | 894 | 784 | 713 | 0 | 0 | Consultation, [7, 8, 9, 10, 11, 12, 13, 14] |
| Diagnostic tests (in monitoring | £/year |  | 5 | 5 | 57 | 57 | 333 | 639 | 0 | 0 | Consultation, [7, 8, 9, 10, 11, 12, 13, 14] |
| Medical research | £/year | 2,134,647 |  |  |  |  |  |  |  |  | [15] |
| Health services paid by government | % | 79.4% |  |  |  |  |  |  |  |  | [16] |
| Health services paid by individuals | % | 15.1% |  |  |  |  |  |  |  |  | [16] |
| Health services paid by other parties | % | 5.5% |  |  |  |  |  |  |  |  | [16] |
| **Productivity costs** |  |  |  |  |  |  |  |  |  |  | [16] |
| Absenteeism | days/year |  | 6.24 | 6.24 | 6.24 | 12.48 | 15.6 | 15.6 | 15.6 | 15.6 | [17, 18, 19] |
| Presenteeism | % |  | -28.3% | -28.3% | -28.3% | -25.6% | -26.7% | -26.7% | -26.7% | -26.7% | [17, 18, 19] |
| Reduced employment | % |  | 0.0% | 0.0% | 0.0% | 0.0% | 0.0% | 40.0% | 40.0% | 40.0% | [20] |
| Proportion of labour force with paid sick leave | % | 100% |  |  |  |  |  |  |  |  | [21] |
| Manager time required per temporary absence | hours | 2.5 |  |  |  |  |  |  |  |  | [22] |
| Manager average hourly wage | £/hour | 33.2 |  |  |  |  |  |  |  |  | [23] |
| Replacement and retraining time | weeks | 26 |  |  |  |  |  |  |  |  | Assumption |
| Years until next turnover | years | 3 |  |  |  |  |  |  |  |  | Assumption |
| Oncosts as a proportion of wages | % | 17.1% |  |  |  |  |  |  |  |  | [24] |
| Discount rate (productivity) | % | 1.6% |  |  |  |  |  |  |  |  | Assumption |
| **Carer costs** |  |  |  |  |  |  |  |  |  |  |  |
| Informal carer time required | hours/week |  | 0 | 0 | 0 | 0 | 0 | 19 | 19 | 19 | [25] |
| NASH population requiring informal care | % |  | 0% | 0% | 0% | 0% | 0% | 45% | 45% | 45% | [26] |
| Formal care costs | £/year |  | 0 | 0 | 0 | 0 | 0 | 96.4 | 97.4 | 186.5 | [26] |
| **Other economic cost** |  |  |  |  |  |  |  |  |  |  |  |
| Average funeral cost | £ | 3,860 |  |  |  |  |  |  |  |  | [27] |
| **Deadweight loss** |  |  |  |  |  |  |  |  |  |  |  |
| Average personal income tax rate | % | 23.4% |  |  |  |  |  |  |  |  | [28] |
| Company tax rate | % | 21.5% |  |  |  |  |  |  |  |  | [29] |
| Deadweight loss | % | 12.0% |  |  |  |  |  |  |  |  | [30] |
| Personal Independence Payment (PIP) | £/year | 3,715.4 |  |  |  |  |  |  |  |  | [31, 32] |
| Number of people with NASH receiving PIP | number | 1,897 | 0 | 0 | 0 | 0 | 0 | 1,747 | 131 | 19 | [31, 32] |
| Employment Support Allowance | £/year | 4,779 |  |  |  |  |  |  |  |  | [33] |
| Number of people with NASH receiving ESA payment | number | 14 | 0 | 0 | 0 | 0 | 0 | 13 | 1 | 0 | [33] |
| Percentage of NASH carers receiving carers allowance | % | 11% |  |  |  |  |  |  |  |  | [34] |
| Payments/year | £/year | 3,359.2 |  |  |  |  |  |  |  |  | [34] |
| **Wellbeing costs** |  |  |  |  |  |  |  |  |  |  |  |
| Disability weight | number |  | 0.000 | 0.000 | 0.000 | 0.000 | 0.000 | 0.178 | 0.296 | 0.000 | [35] |
| VSLY | £ | 60,000 |  |  |  |  |  |  |  |  | [36] |

## Lower probability of diagnosis scenario

Epidemiology results^*^ (lower probability of diagnosis scenario)

|  | **F0** | **F1** | **F2** | **F3** | **F4 CC** | **DCC** | **HCC** | **LT** | **Death** | **Total** |
| --- | --- | --- | --- | --- | --- | --- | --- | --- | --- | --- |
| **Higher scenario (prevalence)** |  |  |  |  |  |  |  |  |  |  |
| Diagnosed (% of prevalence) | 1.0% | 1.0% | 1.0% | 1.0% | 2.0% | 95.0% | 100.0% | 100.0% | 100.0% | 2.0% |
| NASH diagnosed (millions) | - | 0.01 | - | - | - | 0.02 | - | - | 0.01 | 0.04 |
| **Lower scenario (prevalence)** |  |  |  |  |  |  |  |  |  |  |
| Diagnosed (% of prevalence) | 1.0% | 1.0% | 1.0% | 1.0% | 2.0% | 95.0% | 100.0% | 100.0% | 100.0% | 2.0% |
| NASH diagnosed (millions) | - | - | - | - | - | 0.01 | - | - | 0.01 | 0.02 |

*Some results are zero (indicated as -) due to rounding.
F0, Fibrosis stage zero; F1, Fibrosis stage one; F2, Fibrosis stage two; F3, Fibrosis stage three; F4 CC, Fibrosis stage four compensated cirrhosis; DCC, Decompensated cirrhosis; HCC, hepatocellular carcinoma; LT, liver transplant.

Economic costs results (total, £ million) (lower probability of diagnosis scenario)

|  | **F0** | **F1** | **F2** | **F3** | **F4 CC** | **DCC** | **HCC** | **LT** | **Death** | **Total** |
| --- | --- | --- | --- | --- | --- | --- | --- | --- | --- | --- |
| **Higher scenario (prevalence)** |  |  |  |  |  |  |  |  |  |  |
| Health system costs | 1 | 2 | 2 | 2 | 3 | 100 | 10 | 11 | - | 130 |
| Productivity costs | 23 | 37 | 21 | 13 | 17 | 163 | 13 | 2 | 1,421 | 1,711 |
| Carer costs | - | - | - | - | - | 21 | 2 | - | - | 24 |
| Other economic costs | - | - | - | - | - | - | - | - | 18 | 18 |
| Deadweight loss | 5 | 8 | 4 | 3 | 3 | 18 | 1 | 0 | 7 | 51 |
| Total economic costs | **30** | **47** | **27** | **18** | **23** | **302** | **26** | **14** | **1,447** | **1,933** |
| **Lower scenario (prevalence)** |  |  |  |  |  |  |  |  |  |  |
| Health system costs | 1 | 1 | 1 | 1 | 2 | 54 | 5 | 11 | - | 76 |
| Productivity costs | 13 | 20 | 11 | 7 | 9 | 88 | 7 | 2 | 766 | 923 |
| Carer costs | - | - | - | - | - | 12 | 1 | - | - | 13 |
| Other economic costs | - | - | - | - | - | - | - | - | 10 | 10 |
| Deadweight loss | 3 | 4 | 2 | 2 | 2 | 10 | 1 | - | 4 | 28 |
| Total economic costs | **16** | **25** | **15** | **10** | **13** | **163** | **14** | **14** | **780** | **1,050** |

*Some results are zero (indicated as -) due to rounding.
F0, Fibrosis stage zero; F1, Fibrosis stage one; F2, Fibrosis stage two; F3, Fibrosis stage three; F4 CC, Fibrosis stage four compensated cirrhosis; DCC, Decompensated cirrhosis; HCC, hepatocellular carcinoma; LT, liver transplant.

Wellbeing costs results (total, £ million) (lower probability of diagnosis scenario)

|  | **F0** | **F1** | **F2** | **F3** | **F4 CC** | **DCC** | **HCC** | **LT** | **Death** | **Total** |
| --- | --- | --- | --- | --- | --- | --- | --- | --- | --- | --- |
| **Higher scenario (prevalence)** |  |  |  |  |  |  |  |  |  |  |
| Total wellbeing costs | - | - | - | - | - | 189 | 25 | - | 10,250 | 10,464 |
| **Lower scenario (prevalence)** |  |  |  |  |  |  |  |  |  |  |
| Total wellbeing costs | - | - | - | - | - | 102 | 13 | - | 5,525 | 5,640 |

F0, Fibrosis stage zero; F1, Fibrosis stage one; F2, Fibrosis stage two; F3, Fibrosis stage three; F4 CC, Fibrosis stage four compensated cirrhosis; DCC, Decompensated cirrhosis; HCC, hepatocellular carcinoma; LT, liver transplant.

## Higher probability of diagnosis scenario

Epidemiology results^*^ (higher probability of diagnosis scenario)

|  | **F0** | **F1** | **F2** | **F3** | **F4 CC** | **DCC** | **HCC** | **LT** | **Death** | **Total** |
| --- | --- | --- | --- | --- | --- | --- | --- | --- | --- | --- |
| **Higher scenario (prevalence)** |  |  |  |  |  |  |  |  |  |  |
| Diagnosed (% of prevalence) | 10.0% | 15.0% | 16.5% | 58.3% | 80.0% | 100.0% | 90.0% | 100.0% | 100.0% | 25.3% |
| NASH diagnosed (millions) | 0.05 | 0.12 | 0.07 | 0.16 | 0.13 | 0.02 | - | - | 0.01 | 0.54 |
| **Lower scenario (prevalence)** |  |  |  |  |  |  |  |  |  |  |
| Diagnosed (% of prevalence) | 10.0% | 15.0% | 16.5% | 58.3% | 80.0% | 100.0% | 90.0% | 100.0% | 100.0% | 25.3% |
| NASH diagnosed (millions) | 0.03 | 0.06 | 0.04 | 0.09 | 0.07 | 0.01 | - | - | 0.01 | 0.29 |

*Some results are zero (indicated as -) due to rounding.
F0, Fibrosis stage zero; F1, Fibrosis stage one; F2, Fibrosis stage two; F3, Fibrosis stage three; F4 CC, Fibrosis stage four compensated cirrhosis; DCC, Decompensated cirrhosis; HCC, hepatocellular carcinoma; LT, liver transplant.

Economic costs results (total, £ million) (higher probability of diagnosis scenario)

|  | **F0** | **F1** | **F2** | **F3** | **F4 CC** | **DCC** | **HCC** | **LT** | **Death** | **Total** |
| --- | --- | --- | --- | --- | --- | --- | --- | --- | --- | --- |
| **Higher scenario (prevalence)** |  |  |  |  |  |  |  |  |  |  |
| Health system costs | 7 | 16 | 27 | 75 | 102 | 105 | 9 | 11 | - | 351 |
| Productivity costs | 235 | 552 | 342 | 777 | 688 | 171 | 12 | 2 | 1,421 | 4,201 |
| Carer costs | - | - | - | - | - | 23 | 2 | - | - | 25 |
| Other economic costs | - | - | - | - | - | 0 | - | - | 18 | 18 |
| Deadweight loss | 11 | 25 | 16 | 34 | 28 | 4 | - | - | 2 | 119 |
| Total economic costs | **253** | **593** | **385** | **885** | **818** | **303** | **22** | **14** | **1,441** | **4,714** |
| **Lower scenario (prevalence)** |  |  |  |  |  |  |  |  |  |  |
| Health system costs | 4 | 9 | 15 | 40 | 55 | 57 | 5 | 11 | - | 195 |
| Productivity costs | 127 | 298 | 185 | 419 | 371 | 92 | 6 | 2 | 766 | 2,266 |
| Carer costs | - | - | - | - | - | 12 | 1 | - | - | 13 |
| Other economic costs | - | - | - | - | - | - | - | - | 10 | 10 |
| Deadweight loss | 6 | 14 | 8 | 19 | 15 | 2 | - | - | 1 | 65 |
| Total economic costs | **136** | **320** | **208** | **478** | **441** | **163** | **12** | **14** | **777** | **2,549** |

*Some results are zero (indicated as -) due to rounding.
F0, Fibrosis stage zero; F1, Fibrosis stage one; F2, Fibrosis stage two; F3, Fibrosis stage three; F4 CC, Fibrosis stage four compensated cirrhosis; DCC, Decompensated cirrhosis; HCC, hepatocellular carcinoma; LT, liver transplant.

Wellbeing costs results (total, £ million) (higher probability of diagnosis scenario)

|  | **F0** | **F1** | **F2** | **F3** | **F4 CC** | **DCC** | **HCC** | **LT** | **Death** | **Total** |
| --- | --- | --- | --- | --- | --- | --- | --- | --- | --- | --- |
| **Higher scenario (prevalence)** |  |  |  |  |  |  |  |  |  |  |
| Total wellbeing costs | - | - | - | - | - | 199 | 22 | - | 10,250 | 10,471 |
| **Lower scenario (prevalence)** |  |  |  |  |  |  |  |  |  |  |
| Total wellbeing costs | - | - | - | - | - | 107 | 12 | - | 5,525 | 5,644 |

F0, Fibrosis stage zero; F1, Fibrosis stage one; F2, Fibrosis stage two; F3, Fibrosis stage three; F4 CC, Fibrosis stage four compensated cirrhosis; DCC, Decompensated cirrhosis; HCC, hepatocellular carcinoma; LT, liver transplant.

**References**

[1] Estes, C.: Modeling NAFLD disease burden in China, France, Germany, Italy, Japan, Spain, United Kingdom, and United States for the period 2016-2030. Hepatol. 69, 896-904 (2018)

[2] Younossi, Z.M.: The economic and clinical burden of nonalcoholic fatty liver disease in the United States and Europe. Hepatol. 64, 1577-1586 (2016)

[3] Estes, C.: Modeling the Epidemic of Nonalcoholic Fatty Liver Disease Demonstrates an Exponential Increase in Burden of Disease. Hepatol. 67, 123-133 (2018)

[4] Lazo, M.: Prevalence of Nonalcoholic Fatty Liver Disease in the United States: The Third National Health and Nutrition Examination Survey, 1988–1994. Am J Epidemiol. 178, 38-45 (2013)

[5] National Health Service (NHS).: Annual Report on Liver Transplantation. National Health Service (NHS). https://nhsbtdbe.blob.core.windows.net/umbraco-assets-corp/12250/nhsbt-liver-transplantation-annual-report-2017-2018.pdf (2018) Accessed 19 September 2018

[6] National Health Service (NHS).: Annual Report on Liver Transplantation. National Health Service (NHS). https://nhsbtdbe.blob.core.windows.net/umbraco-assets-corp/5007/annual_liver_transplantation_report_2017.pdf (2017) Accessed 19 September 2018

[7] Harman, D.J.: Direct targeting of risk factors significantly increases the detection of liver cirrhosis in primary care: a cross-sectional diagnostic study utilising transient elastography. BMJ Open. 5, 1-10 (2015)

[8] Tanajewski, L.: Economic evaluation of a community-based diagnostic pathway to stratify adults for non-alcoholic fatty liver disease: a Markov model informed by a feasibility study. BMJ Open. 7, 1-11 (2017)

[9] Crossan, C.: Cost-effectiveness of non-invasive methods for assessment and monitoring of liver fibrosis and cirrhosis in patients with chronic liver disease: Systematic review and economic evaluation. Health Technol Assess. 19, 1-410 (2015)

[10] National Health Service (NHS) Improvement.: National tariff payment system 2017/18. National Health Service (NHS). https://improvement.nhs.uk/resources/national-tariff-1719/] (2018) Accessed 27 September 2018

[11] National Health Service (NHS) Improvement.: National tariff payment system 2016/17. National Health Service (NHS). https://improvement.nhs.uk/resources/national-tariff-payment-system-201617/ (2017) Accessed 27 September 2018

[12] National Health Service (NHS) Improvement.: Reference costs. National Health Service (NHS). https://improvement.nhs.uk/resources/reference-costs/ (2018) Accessed 27 September 2018

[13] Curtis, L.: Unit Costs of Health and Social Care 2017., Personal Social Services Research Unit. University of Kent, Canterbury (2017)

[14] Joint Formulary Committee.: British National Formulary 73. BMJ Group & Pharmaceutical Press, London (2017)

[15] Medical Research Council.: The Gateway to Research. UK Research and Innovation. https://gtr.ukri.org/ (2017) Accessed 17 September 2018

[16] Office for National Statistics (ONS).: Health Accounts. Gov.UK. https://www.ons.gov.uk/releases/ukhealthaccounts2016 (2016) Accessed 7 September 2018

[17] Anderson, P.: Real-world physician and patient behaviour across countries: Disease-Specific Programmes - a means to understand. Curr Med Res Opin 2008 Nov;24(11):3063-72.

[18] Babineaux, S.M.: Evidence for validity of a national physician and patient-reported, cross-sectional survey in China and UK: the Disease Specific Programme. BMJ Open 2016 Aug 16;6(8):e010352.

[19] Reilly, M.C.:. The validity and reproducibility of a work productivity and activity impairment instrument. PharmacoEconomics 1993;4(5):353-65

[20] Stepanova, M.: Direct and Indirect Economic Burden of Chronic Liver Disease in the United States. Clin Gastroenterol Hepatol. 15, 759-766 (2017)

[21] Office for National Statistics (ONS).: Sickness absence falls to the lowest rate on record. Gov.UK. https://www.ons.gov.uk/employmentandlabourmarket/peopleinwork/employmentandemployeetypes/articles/sicknessabsencefallstothelowestratein24years/2018-07-30 (2018) Accessed 25 September 2018

[22] Risk Solutions.: The costs to Britain of workplace injuries and work related ill health in 2006/07: Workplace fatalities and self-reports. Health and Safety Executive (HSE). Health and Safety Executive.: The costs to Britain of workplace injuries and work related ill health in 2006/07: Workplace fatalities and self-reports. Risk Solutions. Warrington, England (2011) (2011) Accessed 25 September 2018

[23] Office for National Statistics (ONS).: Population Estimates. Gov.UK. https://www.ons.gov.uk/peoplepopulationandcommunity/populationandmigration/populationestimates (2018) Accessed 11 September 2018

[24] Eurostat.: Labour Costs Annual Data – NACE Rev. 2. European Commission. https://ec.europa.eu/eurostat/web/products-datasets/-/tps00173 (2017) Accessed 8 October2018

[25] Department of Work and Pensions.: Family Resources Survey. Gov.UK . https://www.gov.uk/government/collections/family-resources-survey--2 (2018) 3 October 2018

[26] Scalone, L.: The societal burden of chronic liver diseases: results from the COME study. BMJ Open Gastroenterol. 2, 1-13 (2015)

[27] Royal London.: A False Dawn: Funeral costs rise again after a one year respite. Royal London. https://www.royallondon.com/Documents/PDFs/2017/Royal-London-National-Funeral-Cost-Index-2017.pdf (2017) A3cessed 13 September 2018

[28] Organisation for Economic Co-operation and Development (OECD).: OECD Consumption Tax Trends 2016 – the United Kingdom. Organisation for Economic Co-operation and Development (OECD). https://www.oecd.org/tax/consumption/consumption-tax-trends-united-kingdom.pdf (2016) Accessed 8 October 2018

[29] Spengel, C.: Project for the EU Commission: TAXUD/2013/CC/120: Intermediate Report 2015. Zentrum fur Europaische: Centre for European Economic Research. European Commission. https://ec.europa.eu/taxation_customs/sites/taxation/files/final_report_2016_taxud.pdf (2015) Accessed 28 September 2018

[30] Kleuven, H.: The marginal cost of public funds: Hours of work versus labor force participation, J Public Econ. 90, 1955-1973 (2006)

[31] Department of Work and Pensions.: Personal Independence Payment. Gov.UK. https://www.gov.uk/pip (2018) Accessed 3 October 2018

[32] National Health Service (NHS).: Atlas of Variation In Healthcare for People with Liver Disease. National Health Service (NHS). https://fingertips.phe.org.uk/documents/Atlas_2013%20Liver%20Disease.pdf (2013) Accessed 5 October 2018

[33] Department of Work and Pensions.: Employment and Support Allowance. Gov.UK. https://www.gov.uk/employment-support-allowance (2018) Accessed 4 October 2018

[34] Department of Work and Pensions.: Carer's Allowance. Gov.UK. https://www.gov.uk/carers-allowance (2018) Accessed 4 October 2018

[35] Institute for Health Metrics (IHME).: Global Burden of Disease Study 2016 (GBD 2016) Disability Weights. Institute for Health Metrics (IHME). http://ghdx.healthdata.org/record/global-burden-disease-study-2016-gbd-2016-disability-weights (2016) Accessed 18 September 2018

[36] HM Treasury.: The Green Book. Gov.UK . https://www.gov.uk/government/uploads/system/uploads/attachment_data/file/685903/The_Green_Book.pdf (2018) Accessed 21 September 2018
